# A Practical Treatise on Diseases of Children

**Published:** 1858-03

**Authors:** 


NORTH AMERICAN

MEDICO-CHIRURGICAL REVIEW.

MARCH, 1858.

### Analytical and Critical Reviews.

ART. I.—*A Practical Treatise on the Diseases of Children.* By J. FORSYTH MEIGS, M.D., Fellow of the College of Physicians; Member of the American Philosophical Society, and of the Academy of Natural Sciences of Philadelphia. Third edition, carefully revised. Philadelphia: Lindsay & Blakiston, 1858.

NINE months before the issue of the volume above named, the second edition was exhausted, and we have now before us a third edition, greatly improved. Dr. Meigs's work requires, therefore, no further introduction at our hands; and will be welcome, in its amended form, to those whose appreciation of its value is shown in the ready sale of the preceding editions.

Amid the subdivision of medical labor—which is a growing indication of our progress—it is easy to see why the diseases of children have come to be regarded as worthy of special study, and of separate record. From the epoch of puberty, and the close of childhood, up to old age and death, the adult man, though he may change as to constitution, strength, and endurance of certain remedies, does not undergo those revolutionary alterations which belong to the whole fertile life of the woman, and, in other forms, to that of the child also. The utmost we can say of the adult male is that he is more subject to certain diseases at one age than he is at another. Thus, between fifteen and twenty-five, he is most liable to typhoid fever. Typhus finds him a readier prey after the age of twenty-five. He has also his era of maximum liability to variola, etc. While, however, this is the case, no coincident physiological phenomena mark out these periods in the healthy man; and if such changes do occur, we have no normal notice of their being, and can only infer from our tables of mortality that at such and such a time some change in his economy has made him a readier victim to this or that disease. How different the case in woman is well enough known. After the establishment of puberty she has, besides her menstrual flow, the chances of pregnancy and the final loss of the reproductive capacity to look forward to as, at least in many cases, the causes or occasions of disease. In revolutions of the physiological as of the social state, whatever there is of ill in our corporal community of organs is very apt to find in these eras of change the occasion for development of mischief which unhappily too often survives the period which allowed its birth. Still more remarkable are these times of danger and of change in the growing child. Not only has it constantly to provide for the losses of tissue which result from functional activity, but it is also called upon for the added supplies which are essential to its development toward maturity. Here are nutritive wants, varied in kind, and quite peculiar to the child.

The revolutionary eras of the period of growth are well marked, and offer many diagnostic indications, and many chances for disease which are no longer present in the completed adult. We have thus in the infant a period immediately following birth, when, for a week or ten days, the child either does not gain in weight or actually loses weight. During this time, the secretory organs are active, the accumulated fœces escape, and the child has peculiar liabilities to disease, which have been studied as the diseases of the newly born, and of which we find our author speaking in the proper place. We have next an era of rapid growth, and then the months of teething. This formation of teeth seems to be an indication for their use—in other words, for a change of food. Usually, with us, the time of lactation is a year, and often before the end of it the child is fitted by nature for other and more varied food. Among the lower classes especially, the child is kept at the breast for a much longer time. This is due, as is well known, to an apprehension by the maternal community of the convenient fact of the frequent insusceptibility to reproduction thus obtained. So far as we have seen, it is less hurtful to the child than to the mother, because the child is usually supplied with other and additional food. The second summer is, in this country, another period of danger to the child. Finally, we have the era of second dentition, less fertile of danger, it is true, but still to be remembered; and, lastly, we come to puberty—the boundary between childhood and adult life.

During all these periods of existence, it is curious to note how little the two sexes differ; and to see how, as they draw near to the time of sexual perfection, their lines of life diverge, and the child, which is either male or female, becomes girl or boy, and glides from an almost unsexual neutrality into virile or feminine completeness. In glancing through the preliminary chapter of our author's work, which is devoted to the diagnosis of the diseases of children, we have found a great deal which must interest all physicians; but we were a little disappointed at not finding some more extensive essay in the direction we have just pointed out. A sketch of the physiological life of the child would not have been any more out of place than details as to its respiration and pulse. We may add, that some such special remarks upon the dangerous periods of childhood would be of great use to the student and younger physician, who is more prone to overlook the consideration of these eras than to neglect any other of the diagnostic or therapeutical indications. We hope to see, in a future edition, this physiological sketch of the period of growth. The materials for it are abundant, and, although scattered, would well repay collection, and would fitly preface so good a book as the one we are about to consider.

With the slight exception above stated, we can commend the opening chapter as one of great use and value. For the young medical man, its hopeful tone is also an encouraging reception at the commencement of a volume on a subject which he finds every day of the utmost difficulty. He will here find, well detailed, each point of clinical value in connection with every portion of the body which has the power to aid, by its diseased expressions, in giving certainty to diagnosis. At first sight, what more hopeless cases can be offered to a young doctor than the nursery orchestra of sick and crying children, who disappear under bed and table as the young cutter of gums and tyrant of doses enters the room? As Dr. Meigs assures us, however, these little animals, so dumb as to all power of intelligible lingual expression, are forced to confess their ailments in many ways by disease itself. Unlike the adult, the young child restrains no feeling—has no hysteria, thank heaven, and is honest in disease. Moreover, the babe at the breast has a fixed diet, easily examined—no small advantage in some cases. Lastly, the bawling little patient is open to many ways of questioning. Its heart talks to you in its own fashion. Its breathing speaks, and often plainly enough. Its chest responds to the queries of percussion. Its very cries have meaning, when once is learned this Sanscrit tongue of the nursery; and, finally, the face of childhood is the very play-ground of expression. To all of this Dr. Meigs calls attention, and it is, after all, this kind of knowledge which makes up the most of what is termed experience.

The book under review divides the maladies of childhood into six classes :—1st. Diseases of Respiration. 2d. Diseases of the Digestive Organs. 3d. Diseases of the Nervous System. 4th. Eruptive Fevers. 5th. Diseases of the Skin. 6th. Worms in the Alimentary Canal. Here is no word about the kidney, or the genito-urinary apparatus. It may indeed be said that, for reasons too plain for relation, the procreative organs of the child are but little subject to disease, or only liable, at most, to maladies which claim the surgeon's care. On these accounts we are not often called upon to treat in children diseases of these parts, and we have, therefore, but little mention of them in the work of our author, and of others. While admitting such excuses, we cannot but think that incontinence of urine is a subject for discussion in a work on the diseases of the young. Certainly there is no one of the non-fatal maladies of youth which, in some cases, is more baffling than this, and we believe that the experience of those who have the medical charge of children's homes, houses of refuge, etc., will bear us out in this statement. The mention of it also reminds us that, except in cases of albuminuria after scarlatina, Dr. Meigs does not speak at all of the occasional necessity of examining the urine of the child. In some instances of incontinence of urine such an investigation is essential to a full diagnostic view of the problem before us. In older children the secretion is of course collected as in grown persons. In the infant we have been obliged, where this examination was needed, to direct the nurse to bind a soft caoutchouc bottle, with a large orifice, over the genital organs, by which means, with a little care, enough of urine may be readily collected for purposes of testing and inspection. Another class of cases very frequently demands an examination of the renal excrements. We allude to the dyspepsia of an acid character with which children at the breast are sometimes affected, and which, in them and in those of an older growth, not unfrequently produces uric acid gravel, and, more rarely, permanent calculous affections. The appearance of these deposits in the urine of children we have found to be far more common than is usually supposed to be the case. In this view we are supported by the statements of Dr. Prout, who tells us that of twelve hundred and fifty-six patients received into the Bristol, Leeds, and Norwich hospitals, for the purpose of being operated on for stone, five hundred, or nearly forty per cent., were under ten years of age. If we bear in mind, adds Dr. West, the intimate connection that subsists between the assimilative and the excretory functions, it will not surprise us that in early life, when the former, though so active, are so readily disturbed, the latter should be often thrown into disorder.

As we have stated above, Dr. Meigs begins his list of diseases with those of the respiratory organs. His first chapter treats of coryza, and is a fair and well-written essay upon a malady which, although usually of but little moment, is sometimes of tedious duration. He dwells particularly upon the physiological difficulty which the young child experiences in breathing through the mouth when the nostrils are completely clogged with pus or mucous crusts. In such cases death may result from asphyxia, as in the instance described as an illustration of the severer forms of this disease. On one occasion, several years ago, we encountered a similar case, which resisted all the usual means, such as blowing into the nostrils, caustic applications, etc. The difficulty of breathing through the mouth for any length of time seemed insurmountable. When, at last, satisfied as to the probable result if no further aid were given, we introduced a piece of gum catheter into one nostril, and, after some effort, carried it back into the fauces. Through this the nurse blew occasionally, so as to free the inner end from mucus, and through it the child breathed with evident relief until the other nostril was restored to a healthy state by superficial scarifications and the use of caustics. The means we have mentioned certainly seemed to us to have been effective in aiding, and, perhaps, in saving the little patient.

Our author next treats of the various forms of laryngitis, rightly separating spasmodic croup from pseudo-membranous laryngitis.

The latter, true croup, claims a large share of attention. After insisting upon the absolute necessity of early and vigorous treatment, Dr. Meigs proceeds to discuss the various means to be employed for this purpose. In one hundred and nine cases of spasmodic croup he saw no death, while of twenty-two cases of true croup but ten proved fatal,—an extremely good result, and contrasting very well with the statement of M. Guersent, who cites the mortality of his own cases as eight in ten. Probably he had to deal with hospital cases only, while our author's more agreeable experience is confined to private practice.

Dr. Meigs seems indisposed to bleed, as a general practice, in cases of croup. As he states, (p. 97,) emetics undoubtedly give more relief at the time, and bleeding is to be used, as in other cases, only where the constitution is vigorous and the fever high. Indeed, emetics are regarded by Dr. Meigs as the most efficient remedies in croup. He prefers, on the whole, an emetic proposed by Professor Meigs. It is powdered alum, diffused in molasses, and given in teaspoonful doses. We also have used this article, and can speak confidently as to its great efficiency and certainty. A single dose almost immediately vomits the child, and is never so prostrating as the favorite antimonial preparations. Mercury holds its usual place in our author's catalogue of the means of cure. The use of nitrate of silver, as an application to the pharynx, is also discussed; but our author adds no new facts in regard to its application to the interior of the larynx, although he agrees with Drs. Green and West, in believing such applications possible. The question in regard to tracheotomy in croup receives a full, and we think a satisfactory answer from our author. Dr. Meigs's personal experience in the results of the operation has not been large, but he has collected a mass of information which makes it very clear to us, that not only is the operation justified in many cases, but that where the usual remedies have failed and death seems highly probable, it is a means which should be resorted to without hesitation. It is also very clearly shown that since the introduction of M. Trousseau's improvements, the mortality after the operation has considerably diminished. We regret that we are unable to follow more closely our author's reasonings, and the facts upon which he rests his conclusions in favor of the operation, in cases where calomel, emetics, bleeding, and counter-irritation have failed to bring about a cure.

Congenital and post-natal atelectasis pulmonum are next discussed, and we are offered a very good summary of the present state of knowledge upon this comparatively novel subject. But as it is plainly impossible here to analyze, or review fully the many chapters of Dr. Meigs's work, we prefer, on the whole, to glance at one or two, and then to devote a larger space to the consideration of his views upon scarlet fever and its sequelæ.

As regards hooping-cough, we observe that Dr. Meigs thinks alum, carbonate of potassa, and belladona, the best remedial means. He does not allude to the use of coffee, or to the application of caustics to the pharynx and larynx, as advised by Dr. Brown-Séquard, and ably defended by him upon the ground of the success which he claims to have met with by its use, and also for physiological reasons, which certainly appear to give a color of rationality to its employment.

Under the heading of Diseases of the Digestive Organs, Dr. Meigs occupies one hundred and forty-five pages with the consideration of these maladies. The articles upon diarrhœa, dysentery, and cholera infantum, are perhaps overloaded with numerous opinions of writers upon the value of the remedial means in use, and the summary and criticism of the author upon their views is by no means so good as the corresponding portions of other chapters in the preceding portion of the volume. We find, however, especially in the article on cholera infantum, an attention to the peculiarities of the disease in this country, which is every way commendable, and we cannot but thank Dr. Meigs for his severe strictures upon the criminal carelessness, and even folly, of the parents of American children. We admit, most freely, that climate and other unknown agencies have gone far to modify the constitutions of the human family on this side of the ocean. The feet and hands have become smaller; the voice has a higher note; the face has grown more sallow; the teeth defective, and the features sharper; so that, beyond a doubt, the race is by degrees becoming altered and acclimated. Yet, while nature is doing something to disturb, the loose and wayward habits of our people are also operative, and the whole blame of the frequent ill-health of children, particularly among the better classes, must by no means rest alone upon natural causes. Among the dwellers in towns, the physical education of the young, especially of girls, is utterly neglected. Their dress, also, is often absurd; their diet ridiculously luxurious; and after all this, when the heat of summer or the cold of winter affects them, the blame is laid upon our unlucky climate.

In turning from the class of maladies just alluded to, we meet with some general remarks, which precede the consideration of the diseases of the nervous system. Here Dr. Meigs takes occasion to comment upon the prevailing belief that these disorders occasion a larger number of deaths in childhood than do any others. He believes this opinion to be ill-founded. In this city, between the years 1844 and 1848 inclusive, the number of deaths from diseases of the nervons system was less than from those of the digestive system, and not much greater than from those of the respiratory organs. Practically, however, as M. Barrier hints, the popular view is not so far wrong as would at first appear; we mean to say that it points practically to the fact that the brain of the young is more susceptible of diseased impression than that of the adult,—a point upon which the death statistics cannot tell the whole truth. Thus, every one knows how many children who die from scarlet fever die through the brain, if we may be allowed so to state it. Even in intestinal diseases this is frequently the case; and yet, as a matter of course, none of these are set down as nervous diseases. The physician properly goes beyond the final cause, and past the organ through whose lesion the death occurs, to state in his certificate the name of the disease or the remotely active cause of death.

Passing by the subject of nervous disorders, which are very well treated, and illustrated with some curious and interesting cases, we next encounter Class IV.—*Eruptive Fevers.* We shall here take occasion to dwell upon the scourge of childhood—scarlatina. In regard to the name of this dreaded malady, a popular idea exists that the term scarlatina means a disease of a lighter character than scarlet fever. This is due to the fact that we have no distinctive popular names for the milder forms, and that physicians are sometimes willing to calm the fears of the mother by using the Latin synonym in place of the more familiar English designation. This fearful malady seems to have been quite unknown to the ancients. Some traces of it are to be found in the medical writings of the 16th century, (Ingrassias, 1500,) and it is said to have been imported into Spain from Africa, about 1610. It was described, in 1650, by Martianus, and in 1689

it was seen in London, and delineated by Morton and Sydenham. At and after this time it was more or less confounded with measles, and received a variety of names, which added further to the difficulty of its discrimination. For Sydenham, it was scarlatina; for Morton, morbilli confluentes; for Hoffman, rubeola rosalia; it was the malignant sore throat of Huxham, the febris rubra of Hiberden, and the purpura of Schultz and Jencke. About 1778, Withering finally separated it from measles, and the two are now no longer confounded. Dr. Meigs seems to have had some trouble in his divisions of scarlatina. In this edition he finally rejects them all, and treats of it as one fever, discriminating only as to mild and grave cases. We are not disposed to quarrel with his divisions. They are, after all, equivalent to those of all authors who reject the simple variety without sore throat, and speak of scarlet fever as anginose and malignant only.

Dr. Meigs believes that scarlet fever is propagated by epidemic influence, *i.e.* an aërial poison, and also by contagion. He has seen a child attacked who had never been out of the house at all, it being winter, and the child but five months old. A sister of the child sickened two days later, and a second sister on the third day. Direct exposure, in one sense, was certainly absent in the first-named instance, and our author does not state whether there existed any chance of the conveyance of the poison in fomites, as assuredly does sometimes happen. Thus, in one case, we have known the poison to be conveyed in a garment which was borrowed by Mrs. H. from a lady, in whose family scarlet fever existed, unknown to any one but the family and the doctor. In a second, and very singular example, Mr. E., and his son, aged eighteen years, bought some cakes in the street from a poor woman, who stated that she had made them to sell in order that she might collect a little money to bury her child. The two gentlemen eat of the cakes, and both fell ill of scarlatina nine days after. On strict investigation, it became known that the cakes were made and cooked in the room where the vendor's child had sickened, and then lay dead of a malignant attack of scarlet fever. We are inclined, let us add, to agree with Dr. Meigs in his view as to the possibility of this disease being contracted in either of the ways alluded to, but we also think that every case of supposed propagation by a generally diffused atmospheric influence should be scrupulously criticised.

In connection with this subject is another of much interest, but of which our author does not treat. It very often happens that those who wait upon bad cases of scarlatina become affected with sore throat, and sometimes with a coexistent fever of a few days' duration. We know physicians who are thus troubled whenever they have to attend upon malignant cases. Now, it appears to us possible that this may be in reality a modified form of the fever, occurring in protected or partially protected persons, and resembling thus the false vaccinations which are seen in many persons already vaccinated. However this may be, it is quite clear that such sore throats are not uncommon among the adult attendants of scarlatinal cases. We presume that they will be regarded by many as due to the direct effects of a poisoned atmosphere; but against this there is much to be said, and for it very little, save that the sore throat here described comes on within a period far short of the usual nine days' incubation of the typical scarlatina itself.

A still closer ally of scarlet fever is the scarlatina sinè exanthemata, which is spoken of by many authors. In this form there is no eruption, but we still have itching and desquamation of the skin, as well as sore throat. Dr. Meigs does not allude to this supposed variety, and perhaps rightly, for it is chiefly described by older authors, as Huxham, Fothergill, Rumsey, Lettsom, etc., and is very often, if not always, a scarlatina with a transient eruption. In fact, we once saw a case where the eruption, and that a slight one, was visible but for half an hour, although the attack identified itself as scarlet fever by terminating in dropsy and disease of the kidneys.

Before taking leave of the subject of contagion, we must add that many attempts have been made to innoculate this disease. The efforts at the Orphan House, in Charleston, in 1839, failed entirely, and Petit Radel succeeded no better. Andral states that Stoll was more fortunate; and J. Frank is said to have effected innoculation in dogs; but we have been unable to find their cases, and certainly canine scarlatina seems to us a sufficiently droll idea.

Dr. Meigs thinks that second attacks are more common than is at present supposed. He has seen three examples himself; whereas it is said that scarlatina recurs not oftener than once in a thousand cases. How many were seen in all, by Dr. Meigs, he does not tell us—not a thousand, we presume. It is a little curious that while most physicians here have seen one or more of these second attacks, Willan met with none in two thousand cases; Rayer has seen but one; Dr. Wood, of Edinburgh, in 1839, met with five instances of second attacks, out of only forty-five cases; and Mr. C. Sidney, in the same epidemic, encountered a number of such repetitions.

The question of the relative liability of the two sexes is one which, like almost every point in connection with the subject of contagion, is full of interest, and always difficult of explanation or answer. On the whole, it is best, we think, to view together the double query of liability as modified by age and sex.

Dr. Meigs thinks that scarlatina is most common between the ages of one and five years. Here, as in measles, the period of lactation seems to offer a partial exemption from attack. From this time onward, the two sexes suffer nearly equally, until the approach of puberty, and the period which follows it, when females appear to be more liable than males. This is scarcely a fair inference, although correct enough as a statement of a mere fact. Thus, of some two hundred cases, the proportion of attacks between the ages of six and ten was as seven males to eight females—but a small preponderance. Of the whole number diseased, however, one hundred and thirty-eight were females, and sixty-eight males. Thus, as we have said, the female is more liable after puberty than the male—a circumstance which is probably due to the greater exposure of women, into whose hands the nursing care of scarlatina falls, and who are, therefore, so to speak, in the very focus of infection.

Dr. Meigs next proceeds to distinguish between mild and grave forms. From two hundred and six cases recorded by himself, and from the facts supplied by others, he has given a description of the mild cases, which is very well done, and is a good portrait of the lighter form of the malady. Of grave cases, Dr. Meigs has noted fifty-seven—this class, of course, including scarlatina anginosa and scarlatina maligna. But as his account of the malignant variety possesses no new features, we shall pass on to a review of the sequelæ and complications.

"Dropsy," says our author, "is one of the most frequent and important sequelæ of the disease. It occurred in one-fifth of the cases of MM. Rilliet and Barthez, and in twenty-nine of the two hundred and sixty-three, or, in about a ninth, of those observed by myself."

This sequel of scarlatina is more common in some epidemics than in others. The last epidemic in this city was remarkable for the number of cases of this kind. We saw, for example, eight cases of scarlatina in the spring and early summer months of last year. Of these, four were dropsical. In one of these—already alluded to elsewhere—the eruption was visible for but half an hour, and there was no perceptible desquamation. The œdema of the face was noticed on the twenty-ninth day, and was followed by violent convulsions, which yielded to compression of the carotid arteries, after the warm bath, etc. had failed to be of use. The child recovered. Two of the remaining cases took place in one family, and all finally got well. In another instance, which we met with in 1855, the urine continued albuminous for six months, and until the child had been taken to the seashore. We believe that it is not rare to find the urine thus albuminous for some time after œdema is no longer perceptible.

Another point of interest in this connection, is the cause of the dropsy. Until a year ago, we were altogether disposed to think the dropsy a necessary result of the congestion of the kidneys, which always accompanies it. Since then, however, we have been in the habit of examining the urine every day from the time the disease declares itself, and we have twice observed the water albuminous before the least œdema was to be seen. In one case, the albumen was detectable on the twelfth day, while the œdema could only be observed on the fifteenth, and continued but four days—the urine remaining albuminous and slightly bloody for a much longer time.

We have also seen cases—two in number—where there was local dropsy—ascites—without albuminuria. Newbigging (*Ed. Monthly*) states the urine to have been albuminous in every case, and Begbie holds the same opinion. They speak of testing for albumen by heat only. It is scarcely possible that they neglected to employ also nitric acid. If they did not use both tests they may have been misled, since, in scarlet fever, the phosphates will very often be thrown down by heat. Nitric acid redissolves this precipitate, thus showing an absence of albumen. Martin Solon states that of twenty-three cases which he saw, twenty-two were albuminous.

Dr. Meigs's account of the symptoms which accompany or announce the presence of dropsy, is extremely good. As to the cause of the renal accompaniment, he does not undertake to decide; nor does he state here or elsewhere that a similar dropsical complication sometimes, though rarely, is seen in measles and erysipelas. As regards his account of the appearances afforded by the urine in these cases, we have only to add that it is scarcely so full or so complete as it might usefully be.

As a general rule, the urine is dark-brown or muddy, as Dr. Meigs describes it. This statement is true of this fluid at and after the crisis of the renal disease; but if Dr. Meigs will carefully note the state of the secretion every day until, in some instance, he lights upon the first signs of a coming congestion of the kidney, he will find that the urine is usually pale at first, then pink, then red, and finally of a very dark brown. In other words, the congestion of the kidneys comes on by degrees. When, as does sometimes chance, the attack is acute, the urine becomes bloody and deep hued with greater suddenness. From the period of the height of the congestion, the urine gradually becomes less brown and muddy, and deposits a sediment which in time is brown, yellow, ashen, and white, as the amount of blood diminishes. This deposit consists of altered blood-discs, epithelial cells, and fibrinous casts of the renal tubules, which are sometimes covered with blood-discs, and sometimes with epithelia. The presence of these casts is not spoken of by Dr. Meigs, nor does he remark at all upon the interesting and important changes in character which they undergo as the disease progresses or declines. We may also add, that the sediment very rarely contains "lithate of ammonia *in crystals*," as Dr. Meigs states, and that the deposit so called is now commonly considered to be lithate of soda, and to contain very little of the ammonia salt. It also is very rare to find either the one or the other of these compounds in a crystalline state.

Turning from the unpleasant task of noting defects, we are much pleased with our author's views as to the remedial means to be employed in scarlatina. If there is any deficiency here—and this is a matter of individual opinion—it is in regard to the freer use of baths, which we have used, in some cases as frequently as every hour, and which we are also accustomed in some cases to employ daily for three or four weeks. Johnson saw no cases of sequent dropsy, where this means was used. We have seen two, but are yet of opinion that the warm bath, when thus continued, lessens the chances of the secondary malady. On all other matters we are fully agreed with our author, and we believe that his essay well represents the most rational views of treatment.

Inunction, baths, and the use of chlorine, chlorates, ammonia, etc., are the means upon which he mostly relies. His report and criticisms upon the employment of cold baths are every way excellent, and we really regret that they are too long for transfer to our pages. Dr. Meigs does not appear to have used belladonna as a remedy during the disease.

Like our author, we are not prone to deplete in dropsical cases. It is a measure of doubtful value, and the child who has been drained by scarlet fever is not often a fit subject for the leech or the lancet. When the crisis is over, diaphoretics and cathartics are, in most cases, all that we require. In some instances the flow of blood from the kidneys goes on for months, the urine remaining dark or pinkish in hue, and the little patient growing weak and anæmic. Under these circumstances, tinct. ferri. chloridi, gallic acid, change of air, etc., are needed in addition to the usual means treated of by Dr. Meigs. Among these are also diuretics, which our author always employs in these cases, using acetate of potassa, bi-tartrate of potassa, squill, juniper berries, etc. We, also, have made use of these remedies, and in three cases have watched with care their effect upon the congested organs. The secretion was in no instance increased by them to a greater extent than when the quantity of pure water, which had accompanied them in the prescribed draughts, was given alone.

We are sorry that Dr. Meigs is not able to speak, from extensive use, of the value of belladonna as a prophylactic in scarlatina. He has only employed it within four years, and possesses no exact data upon which to form a definite opinion. We are glad to find that he leans to the belief that the evidence in favor of its success is such as to make its employment a matter of duty. We have gone over, with care, a large mass of evidence upon this subject, including many articles written since the publication of Dr. Peyre Porcher's most admirable paper, (*Charleston Med. Journal and Review,*

1851,) and we are still inclined to adhere to the use of belladonna. We had hoped; indeed, to make use of our materials in this review for the purpose of exhibiting how strong the presumptive evidence is in regard to its prophylactic value. Want of time, however, warns us to dismiss the subject thus briefly, and we must, therefore, content ourselves with this simple expression of opinion, founded on considerable reading and some personal experience.

The remaining portion of our author's book deals with skin-diseases and worms. The article upon measles is a well-written account of this disease, and contains certain interesting facts as to the mortality, etc. of the malady in question. Dr. Meigs is of opinion that the rubeola sine catarrho of some authors is mere roseola. He has seen it only in infants. During 1857 it was epidemic in this city, and we saw a number of cases, chiefly among medical students, several of whom had already had measles. It is quite possible that Dr. Meigs may be right; and, at all events, the differential diagnosis between rose rash and the form of measles just mentioned is by no means easy.

The chapters upon small-pox, varioloid, vaccination, and re-vaccination, we have found to be extremely good,—the many mooted points in connection with these topics being fairly discussed, and treated with clearness and good sense. Here, as elsewhere, we are pleased with our author's criticisms and comments upon the indications presented by the disease, and upon the relative values of the remedies in common use.

In taking leave of a book in which we have found some omissions, some defective statements, and much that is excellent and worthy of praise, we feel bound in honor to mention that the author apologizes in his preface for his failure to treat of many points upon which he has collected ample material. Meanwhile, it is plain, from a comparison of this edition with preceding ones, that at every change Dr. Meigs has improved both as a writer and reasoner, so that, without doubt, any future re-issue will give to us the greater part of what the author has here omitted to treat of, or has dealt with insufficiently.

Let us finally thank Dr. Meigs for a sensible book about children. Poor little breeched or petticoated, stuffed, petted, bedizened, or half-naked little creatures, the share of the common sense of the world which is devoted to them and their service is small indeed. Let us, at least, do our best for them. Of parents, in this country, we have but little hope. Dead relatives and children are the luckless means of exhibiting the follies of friends and parents. As papa cannot don the kilt, and show his bare knees on Chestnut Street at 32° Fahrenheit, master Johnny serves to show what papa would like to do. The little women are no better used; and when the little body's dress gets up to the knees and down to the arm-pits, we may feel sure that it is typical of that primitive leaf which once represented the fall fashions, but we cannot concede to it the title of a dress.

As to medicine, and its rational uses among children, what can we say? Who does not know of many families where the quacks of Lilliput (would that Swift had known them) are the ordinary medical attendants of the children, while a physician of regular repute is as invariably called to prescribe for the parents and adults generally? Now this is a fact of very common occurrence, and, in the name of all absurdity, what does it mean? Is not this the very cream of folly? Is it really that the homœopath is supposed to be the next best doctor to nature, because he is thought to do nothing? Poor libelled nature! Or is it believed that little pills and little folks possess some mysterious medical relation?

But, admitting the existence of this singular illustration of maternal folly, when does homœopathy cease to be good for the child? At what age does scientific medicine become suited to the budding man or woman? Is there an era in growth, somewhere, which is suited to both at once, or to neither? Alas! would that, like the disciples of Hahnemann, we possessed remedies against folly and dullness, anger, melancholy, and the like, for then would we deal liberally to the mothers of all the babies everywhere such draughts of common sense as should materially lessen, in future, the profits of our profession.

